# Study of EEG characteristics while solving scientific problems with different mental effort

**DOI:** 10.1038/s41598-021-03321-9

**Published:** 2021-12-10

**Authors:** Yanmei Zhu, Qian Wang, Li Zhang

**Affiliations:** 1 School of Early Childhood Education, Nanjing Xiaozhuang University, Nanjing, China; 2 School of Biological Science & Medical Engineering, Southeast University, Nanjing, China

**Keywords:** Neuroscience, Psychology

## Abstract

Studying the mental effort in problem-solving is important to the understanding of how the brain allocates cognitive resources to process information. The electroencephalogram is a promising physiological approach to assessing the online mental effort. In this study, we investigate the EEG indicators of mental effort while solving scientific problems. By manipulating the complexity of the scientific problem, the level of mental effort also changes. With the increase of mental effort, theta synchronization in the frontal region and lower alpha desynchronization in the parietal and occipital regions significantly increase. Also, upper alpha desynchronization demonstrates a widespread enhancement across the whole brain. According to the functional topography of brain activity in the theta and alpha frequency, our results suggest that the mental effort while solving scientific problems is related to working memory, visuospatial processing, semantic processing and magnitude manipulation. This study suggests the reliability of EEG to evaluate the mental effort in an educational context and provides valuable insights into improving the problem-solving abilities of students in educational practice.

## Introduction

Mental effort represents the quantity of cognitive resources involved in performing a task^1–3^. It is seen as a combination of the perceived demand characteristics, depth of information processing, and personal expertise^1,4^. Perceived demand characteristics mainly depend on the inherent complexity of the task content, which is related to the degree of interaction between various information elements^4^. When element interaction is low, task information can be extracted and learned for one single element at a time since individual elements have minimal references to one another. On the other hand, highly interacting information consists of elements that heavily interrelate, which must be combined and processed simultaneously. Accordingly, the more the number of interacting information a task contains, the higher the cognitive demand and mental effort it elicits^5^. Depth of processing refers to the degree to which a person encodes information. Deeper processing implies a greater degree of semantic or cognitive analysis of input for meaning extraction and comprehension with their existing knowledge, while surface level processing is more concerned with the recognition of physical or sensory features of the stimulus^6^. Thus, deeper processing of presented information requires higher mental effort than a more surface level encoding. Furthermore, developed expertise resulting from repeated practice lowers the mental effort imposed by a familiar task^7,8^. Studying the mental effort in problem-solving helps to determine the online processes while individuals are working on these problems and provides a deep understanding of their learning outcomes^9,10^. It could also provide implications for the development of effective instructions to improve their learning performance. For example, researchers have found that lead-augmented hypertext system improves the learning performance by visualization of interacting information elements as one undivided unit^5^. This system decreases the mental effort required for the integration of the hypertext nodes with semantic space. However, many of the previous studies usually explored the mental effort imposed by well-controlled working memory tasks or other problems which required little scientific knowledge. Compared with these tasks, scientific problems require the abstract scientific conception and involve complex cognitive processes such as model representation, retrieval of scientific conception and inference calculation. Solving scientific problems will involve a set of brain resources and consequently a high level of mental effort. To our knowledge, mental effort during scientific problem solving is still seldom reported. Research on mental effort in scientific problem solving may shed light on scientific reasoning of students and suggest ways to improve their problem-solving ability in science education.

Various methods, including subjective measures, secondary task measures, and physiological measures, have been applied to the assessment of mental effort^11–13^. Subjective measures evaluate the mental effort by using the subjective rating scales^14^. Secondary task measures estimate the mental effort for the primary task according to accuracy and response time on the secondary task^15^. Physiological measures quantify the mental effort by measuring a variety of physiological factors such as heart rate variability, eye movement and brain activity^16–20^. Both subjective and secondary task measures do not allow for a continuous and noninvasive measurement of mental effort. The participant has to interrupt the main task to fill a questionnaire or to perform a secondary task during the main one. In contrast, physiological measures can continuously and objectively monitor online mental effort without interfering with task performance^21,22^. Also, physiological measures vary predictably in response to changes of mental state^23^. It can be used to anticipate a mental impairment such as cognitive overload and fatigue^24,25^. However, through subjective and secondary task measures, due to lack of the capacity to monitor covert changes in psychophysiological state, it is only possible to detect a mental impairment once it already happened. Moreover, some studies have proved that physiological measures provide higher sensitivity in discriminating different levels of mental workload compared to subjective and secondary task measures^26,27^. In these studies, physiological variations reliably represent implicit fluctuations in the mental states, suggesting that physiological measures have sufficient stability and sensitivity to distinguish between different degrees of mental effort.

The electroencephalogram (EEG) is a promising physiological approach to the assessment of the mental effort of students in an educational context^28,29^. It can directly monitor an individual’s cognitive state by recording the electrical signals produced by the brain in an authentic environment. Previous studies have examined changes in EEG signals as a function of mental effort^30–33^. Researchers pointed out that brain activity in the theta and alpha frequency is sensitive to effortful processing^34–37^. This result was found in the studies about the electrophysiological indicators of mental effort. Most studies that apply the well-controlled working memory tasks, such as N-back tasks or spatial and verbal working memory tasks, have reported both alpha and theta effects on cognitive effort. With the enhancement in memory load due to increased task difficulty, an increase in frontal theta power and decrease in parieto-occipital alpha power are usually observed^38–43^. Research using multitasking also revealed a strong association between mental effort and oscillations in the theta and alpha frequency. They found that a growth in the number of subtasks the participants performed simultaneously increased the amount of theta activity in the frontal region and decreased alpha activity in the parietal region^44^. In addition, a similar pattern was found in studies using arithmetic problems or some real-world simulation tasks like air traffic control^28,45^. These studies have shown that changes in the frontal theta and posterior alpha activity are reliable indicators of mental effort elicited by tasks of varying complexity.

Compared with the tasks in the previous work, scientific problems require abstract scientific conception and involve complex cognitive processes. Solving scientific problems activate a set of brain resources and a high level of mental effort. In this case, a refinement of EEG mental effort measure proposed by Smith and Gevins should be considered^46^. Their EEG workload model includes quantification of alpha and theta band activity recorded from the frontal brain area essential for working memory and executive control, centro-parietal area essential for visuospatial processing, occipital area essential for stimulus encoding and semantic memory processing. Changes in EEG-derived regional indices could provide information about the relative activation of different local cortical regions in response to increases in mental effort. Based on these regional indices and specialization of the underlying cortical regions, neurologically meaningful segregation of task effects on mental effort can be speculated. Using this model, Smith and Gevins identified the different levels of mental workload in a flight simulator task. The mental state of an individual, whether in a well-rested state or following a total sleep deprivation, can be determined according to regional indices reflecting both task demands and operator’s state of alertness^46^.

In this study, we attempt to investigate the EEG indicators of mental effort during scientific problem-solving. To this end, we asked students to solve the scientific problems commonly used in the practice of science education while recording their EEG signals. By manipulating the complexity of the scientific problems, students engaged in different levels of mental effort. Their EEG signals during problem-solving were examined. We focused on analyzing the EEG activity in the theta and alpha frequency, because these two bands are particularly important indicators of effortful processing. Besides, brain activity in the lower and upper alpha frequency has been documented to be associated with differential cognitive effects. The lower alpha changes are considered to reflect general task demands, such as attentional processes, while the upper alpha changes are suggested to reflect the task-specific processes such as stimulus encoding, semantic processing and memory access^42^. Although these two alpha bands have different functional specializations, many previous studies employed the entire alpha band to explore EEG indicators of mental effort^44,47,48^. Some studies used simple working memory tasks to calculate the changes in the lower and upper alpha frequency. They found that the two alpha bands seemed to respond quite similarly in simple tasks^49^. Compared with simple tasks, solving scientific problems involves more complex cognitive processes including attention, working memory, visual processing, semantic memory and multisensory integration. We expected that task-general and task-specific processes during scientific problem solving would be reflected by the lower and upper alpha activity, respectively. For this consideration, both lower and upper alpha bands were examined in this study. We hypothesized that functional specialization of these two alpha oscillations would appear in our task requirements. They would reflect the neural responses to mental effort imposed by the particular cognitive processes while solving scientific problems. According to the functional topography for theta and alpha oscillations, we expected that mental effort imposed by general attention would be associated with posterior lower alpha activity, mental effort by working memory and executive function would be reflected by theta and upper alpha activity in the frontal area, mental effort by task-specific processes of visual processing, semantic processing and multisensory integration would be reflected by upper alpha activity in the occipital and centro-parietal areas. Moreover, with the increase of mental effort, theta oscillation would increase while both alpha bands would decrease.

## Results

### Behavioral results

**Figure 1:**
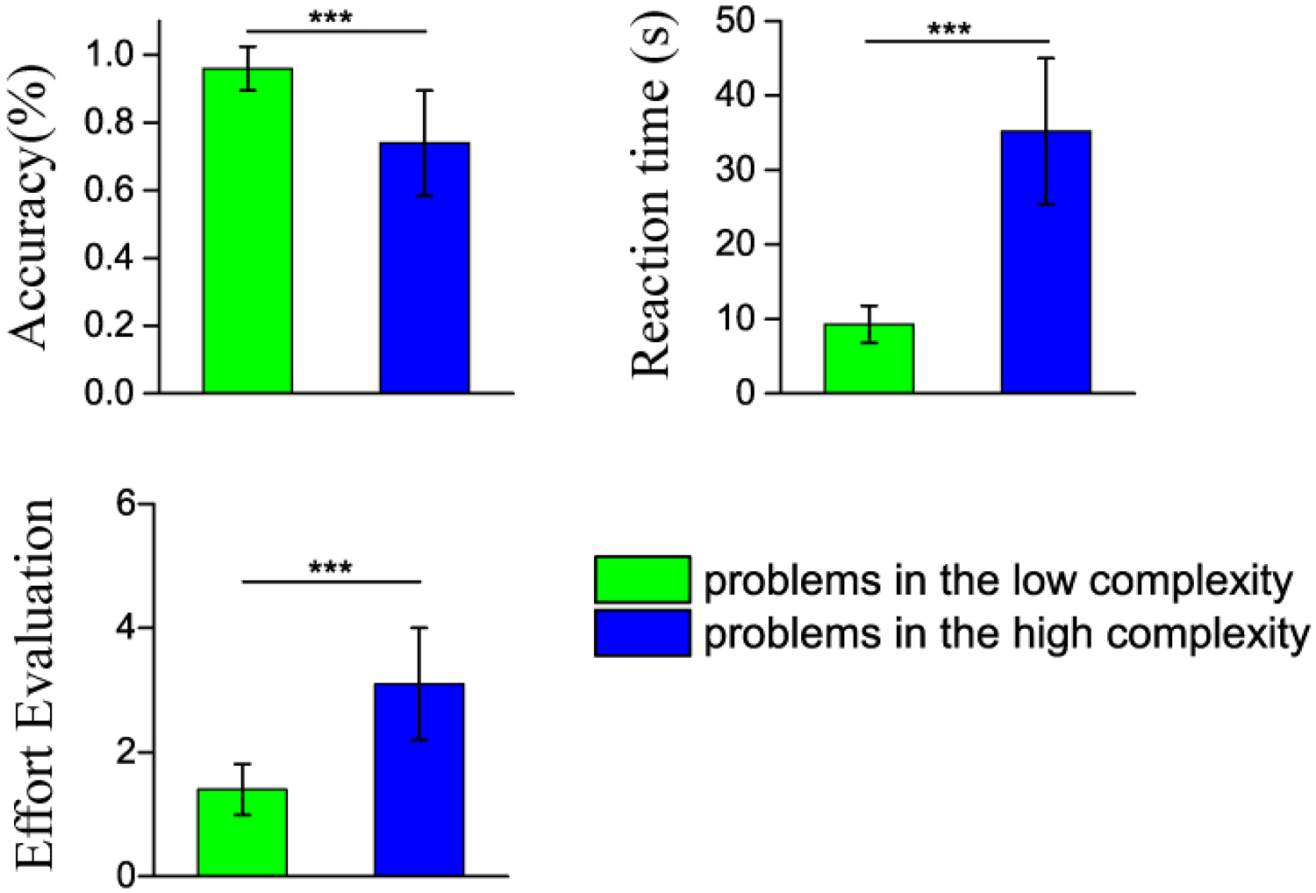
Mean reaction time, response accuracy and subjective effort evaluation for the low and high complexity problems. The behavioral results support that our scientific problem design and complexity categorization are reliable. The experiment successfully engaged the students in solving scientific problems with different levels of mental effort. *** *p* < 0.001.

Mean reaction time, response accuracy and subjective effort evaluation for the low and high complexity problems are shown in Table 1 and Fig. 1. In our study, students gave 9.6 ± 0.6 and 7.4 ± 1.6 correct trials for the low and high complexity problems, respectively. Wilcoxon signed-rank test reveals the main effect of problem complexity condition (*Z* = − 4.37, *p* < 0.001, for reaction time; *Z* = − 4.04, *p* < 0.001, for response accuracy; *Z* = − 4.29, *p* < 0.001, for subjective effort evaluation). The results reflect that high complexity problems are more demanding than the low complexity ones. Students spent more time to solve these problems, but the response accuracy declined significantly. In addition, during the interview session, students responded that these high complexity problems required more effort. All the behavioral results support that our scientific problem design and complexity categorization are reliable. The experiment successfully engaged the students in solving scientific problems with different levels of mental effort.

**Table 1:** Mean reaction time, response accuracy and subjective effort evaluation for the low and high complexity problems.

Problem complexity	Reaction time (s)	Response accuracy (%)	Subjective effort evaluation
Low	9.26 ± 2.60	96.0 ± 6.5	1.4 ± 0.4
High	35.19 ± 13.43	74.4 ± 15.5	3.1 ± 0.9

### Electrophysiological results

**Figure 2:**
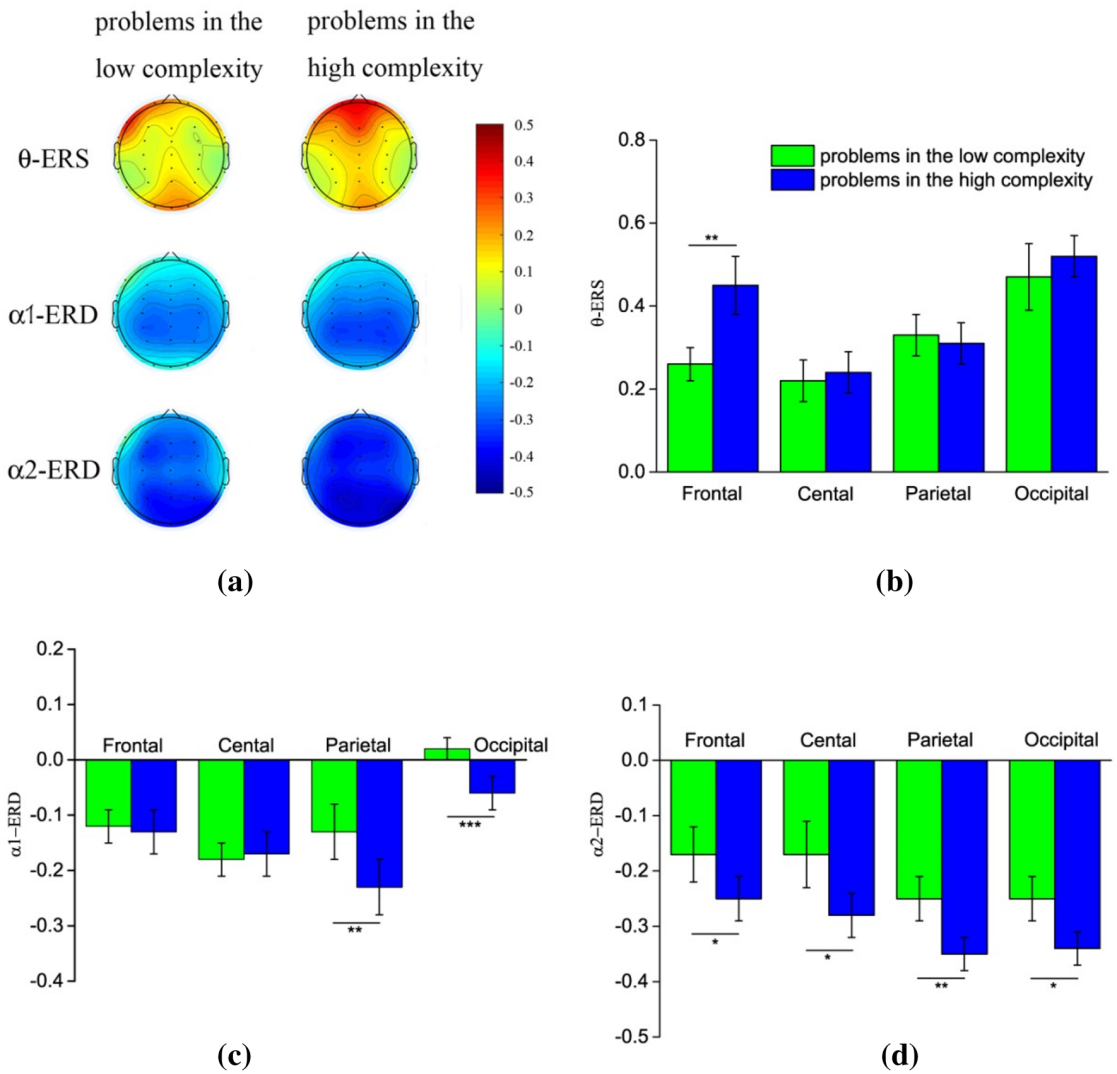
ERS/ERD in the theta and alpha bands while solving the low and high complexity scientific problems. α1 refers to lower alpha band, α2 refers to upper alpha band. With the increase of mental effort, the amplitudes of frontal theta ERS and posterior lower alpha band ERD increase, while the amplitude of upper alpha band ERD demonstrates an extensive enhancement throughout the whole brain. (**a**) Topographical maps of theta ERS and alpha ERD for the two complexity conditions. (**b**) Theta ERS for the two complexity conditions in four brain regions. (**c**) Lower alpha band ERD for the two complexity conditions in four brain regions. (**d**) Upper alpha band ERD for the two complexity conditions in four brain regions.

Figure 2 gives the neurophysiological results. Figure 2a illustrates the topographic maps of theta ERS and alpha ERD for the two conditions of problem complexity. Figure 2b–d gives the statistical results of theta ERS, lower alpha band ERD, and upper alpha band ERD in the different brain regions, respectively. The results indicate that theta activity increases, and alpha activity decrease during scientific problem solving. In addition, the amplitudes of theta ERS and alpha ERD vary across brain regions between the two problem complexity conditions.

For the theta ERS, as shown in Fig. 2b, a two-way repeated measures ANOVA obtains the significant main effect of brain area [*F*(3,72) = 6.98, *p* < 0.01, *ƞ*^2^ = 0.23]. The theta ERS in the frontal and occipital regions are significantly higher than those in the central and parietal regions (both *p* < 0.05). The results also reveal the significant interaction of the brain area and problem complexity condition [*F*(3,72) = 24.13, *p* < 0.001, *ƞ*^2^ = 0.51]. Simple effect analysis reflects that theta ERS tends to be higher for the high complexity scientific problems than the low complex problems in the frontal region ([*F*(1,24) = 14.84, *p* < 0.01]). There is no significant difference in theta ERS for two complexity conditions in other brain regions (all *p* > 0.05).

For the lower alpha band ERD, as shown in Fig. 2c, the statistical results reveal a significant main effect of brain area [*F*(3,72) = 9.08, *p* < 0.001, *ƞ*^2^ = 0.28], with a significantly stronger lower alpha band ERD at the central and parietal regions compared to the frontal and occipital regions (both *p* < 0.05). The results also show a significant interaction of brain area and problem complexity condition [*F*(3,72) = 5.38, *p* < 0.001, *ƞ*^2^ = 0.18]. Simple effect analysis shows a stronger lower alpha ERD in the parietal ([*F*(1,24) = 13.03, *p* < 0.01]) and occipital regions ([*F*(1,24) = 24.58, *p* < 0.001]) when solving the high complexity problems, in comparison to low complexity problems. Lower alpha band ERD has no significant difference between two complexity conditions in other brain regions (all *p* > 0.05).

For the upper alpha band ERD, as shown in Fig. 2d, the results show a significant main effect of problem complexity condition [*F*(1,24) = 6.48, *p* < 0.05, *ƞ*^2^ = 0.21]. Upper alpha band ERD is much stronger for the high complexity problems. The main effect of brain area as well as the interaction of the brain area and problem complexity are both not significant (both *p* > 0.05). This result suggests a significant global suppression of upper alpha activity when solving high complexity scientific problems. Paired-*t* test was conducted on the amplitude of upper alpha band ERD between the low and high complexity condition. The results confirm that upper alpha band ERD is significantly stronger across all brain regions (*p* < 0.05 in the frontal, central and occipital regions; *p* < 0.01 in the parietal region.)

## Discussion

In this study, we investigate the EEG characteristics of different mental effort in solving scientific problems. By manipulating the complexity of the problem, students engaged in tasks with different levels of mental effort. Both behavioral data and interview reports show that they spent more mental effort in dealing with the high complexity scientific problems. The different amounts of mental effort are accompanied by theta ERS and alpha ERD, which exhibit the distinct topographical distribution. Particularly, an increase in mental effort significantly enhances the amplitude of frontal theta ERS, parietal and occipital lower alpha band ERD, and brain-wide upper alpha band ERD. These task-induced EEG changes reflect the mental resources required for the specific cognitive processes involved in solving scientific problems.

### Brain activity in the frontal theta increases while solving scientific problems

Our findings show that frontal theta activity increases with the enhanced mental effort while solving scientific problems. Frontal theta oscillations are usually observed in EEG studies that use working memory tasks, multiple tasks, and other tasks involving executive functions. The results show that frontal theta activity is positively correlated with task demands on memory load and executive function^35,38–40^. Some studies have even found that the degree of theta synchronization is a neural signature of successful information manipulation^50^. Recently, researchers employed the real-world simulation tasks such as flight and air traffic management to investigate the EEG indicators of mental load^25,45^. They also found that as cognitive demands on attention, working memory load and task control increase, the enhanced mental effort leads to an increase in the frontal theta activity. These previous studies revealed that frontal theta synchronization is a reliable indicator of mental effort elicited by working memory and executive function.

Consistent with the previous studies, our results further demonstrate that the frontal theta synchronization becomes stronger as mental effort increases when solving the high complexity scientific problems. It suggests that more working memory and control capacity are allocated to accomplish these problems. This result is reasonable since more information and deeper processing are involved in high complexity scientific problems. These problems involve two or more objects. The forces acting on an object will be affected by others as well as the motion state of the system. Students need to analyze each object and the entire system at the same time. Meanwhile, they need to retrieve the physics concepts and apply them to analyze the forces acting on the objects. More interactive elements and greater control requirements expand the amount of information maintained, manipulated and controlled in working memory. Therefore, more mental effort is recruited. In contrast, for the scientific problems with the low complexity, only one object is involved, and there is no complex interaction between objects. Furthermore, in educational practice, it is common to determine the forces acting on a single object in the system. Fewer information elements and the developed expertise make the reasoning process in working memory more automated, thereby reducing online mental effort of the task. Our results suggest that frontal theta activity can reliably reflect the mental effort assigned to working memory and control processes when solving abstract scientific problems.

### Brain activity in the alpha frequency decreases while solving scientific problems

In our study, both the lower and upper alpha activity decrease with the increase of mental effort while solving scientific problems. Alpha oscillations are usually found to be negatively correlated with effortful processing^27,42^. Alpha desynchronization is suggested to represent an intensified recruitment of relevant mental resources when task demands require more cognitive processing to perform it^51^. Studies on visual or auditory information processing, sensorimotor processing, multitasking and reasoning have proven that the quantification of alpha ERD is a particularly useful and appropriate method for measuring the level and topographical distribution of cortical activation during cognitive task performance. For example, alpha ERD is maximal in the occipital region during the visual task, maximal in the temporal region during the auditory task, and maximal in the centro-parietal region during sensorimotor task^44,52–55^^.^ Moreover, the amplitude of alpha ERD becomes more prominent when task difficulty increases. Besides, brain activity at the lower and upper alpha frequency has been documented to reflect different cognitive demands. Desynchronization of lower alpha has been observed in responses to almost any type of task, and is believed to represent the general task requirements for basic arousal and attentional processing^42^. The ERD map consistently displays that the lower alpha desynchronization is the largest in the parietal area^42,56^. Moreover, it is observed that the lower alpha is highly sensitive to practice^37^. Generally, the amplitude of lower alpha activity increases significantly with the increase of practice, suggesting that performance requires lesser mental resources after the individual has completed the task proficiently. In contrast, desynchronization of upper alpha usually emerges at task-relevant sites and is considered to reflect specific task requirements, such as stimulus encoding, semantic processing and memory access^56–59^. Although these two alpha bands have different functional specializations, many previous studies employed the entire alpha band to explore EEG indicators of mental effort^47,48^. Some studies have calculated changes in the lower and upper alpha bands, but used simple tasks such as working memory tasks. They found that the two alpha bands seem to respond very similarly to mental effort in simple tasks^49^. In our study, considering that more complex cognitive processes are involved in solving scientific problems, we calculated the brain activity in the lower and higher alpha band respectively. These two alpha bands demonstrated the different effects on mental effort allocated by the abstract scientific problems.

In line with the previous studies, we found that sensitivity of online mental effort in the lower alpha band located in the parietal and occipital brain areas during solving scientific problems. According to the cognitive effects of lower alpha activity, ERD in the parietal area suggests that more general alertness and attention are imposed on stimulus representation, while ERD in the occipital region reflects that more visual attention is required for stimulus sensory processing^60,61^. It is reasonable because more complex visual elements are included in these problems. In addition, compared with the progressive automation of single-object problems in educational practice, complex scientific problems still require sustained and focused attention. Accordingly, the greater amplitude of lower alpha band ERD in the posterior region suggests that a higher level of mental effort is required to gain general attention when solving complex scientific problems.

Different from the previous studies which showed similar activation patterns between lower and upper alpha activity due to the application of simple tasks, we observed a distinct topographical distribution of upper alpha band ERD compared to that of lower alpha band. In our study, the upper alpha band ERD is enhanced throughout the whole brain areas with the increase of mental effort. Our results support the view of functional differences between different alpha bands, and are also in line with the statement that the functional specialization of the lower and upper alpha bands is most prominent in the frontal and central regions as the task demands increase^42^.

It is commonly observed that upper alpha band ERD is the largest at occipital site when processing various visual stimuli^56^. In these visual tasks, researchers found that desynchronization started over occipital site in the upper alpha band, followed by a parietal localized ERD in the lower alpha band. They explained that the early upper alpha band ERD at occipital site reflects visual encoding of the stimulus, including feature extraction, stimulus identification, semantic encoding and memory access, while the following parietal ERD in the lower alpha band reflects attention on encoded visual stimulus^56^. Other studies using semantic processing tasks consistently found that ERD in the occipital region is the most sensitive to the search and retrieval processes of semantic information, and is significantly correlated to the performance of semantic memory^57^. These results reflect that upper alpha in the occipital site is indicative of visual stimulus encoding and semantic memory processing. In our study, students not only need to encode the symbolic diagram and make a mental representation of current information, but also need to retrieve the physics conception from long-term memory and integrate scientific law with information presented in the problem. Mental resources for stimulus encoding and semantic memory processing are particularly higher for the complex scientific problem because it involves interactive objects in the diagram and requires deeper processing for meaning extraction and comprehension. We suggest that this heavier mental effort would be reflected by the stronger upper alpha band ERD in the occipital region.

Upper alpha band ERD in the centro-parietal regions is reported to be essential for visuospatial processing and mathematical ability^54^. It is commonly observed that centro-parietal alpha power decreases in tasks with visually presented stimuli. Several studies have demonstrated that generation of ideas in the figural domain is related to the strong decrease in the upper alpha activity at parietal site, which reflect the high visuospatial processing requirements during mental generation and manipulation of visual representation^62,63^. In the field of mathematics, researchers found that the parietal site supports magnitude manipulations in arithmetic tasks^64^. In addition, ERD results revealed that applying procedure strategy to solve more difficult arithmetic problems causes much stronger activation in the parietal regions than using strategy that retrieves answers directly form long-term memory for easier problems^65^. Accordingly, we suggest that the stronger upper alpha band ERD in the centro-parietal region indicates the greater mental effort for visuospatial processing of symbolic diagram in the high complexity problem. Quite similar to the difficult arithmetical problems, complex scientific problems also require a procedure strategy to analyze the forces on each object, rather than directly retrieving the answer from long-term memory. In other words, students need to first analyze the forces based on the motion state of the entire system, and then consider the forces exerted on each object according to its motion state as well as the interaction between adjacent objects. In this case, magnitude manipulations are also involved to calculate and determine the forces due to interaction between objects. The mental effort allocated by these cognitive processes would be reflected by the centro-parietal upper alpha desynchronization.

Frontal upper alpha desynchronization is usually used to examine higher order cognitive functions of executive processes, such as inhibition, and mental manipulation of information^55,66^. These cognitive processes are important for learning, reasoning, and comprehension. Stronger frontal upper alpha band ERD suggests that greater mental effort is allocated by executive function and working memory demands in task performance. Researchers further suggest that upper alpha activity at frontal site reflects the degree of executive control of anterior brain region over more posterior regions responsible for cognitive processes including information encoding, memory tracing, visuospatial processing and other mental operations^55^. For example, the activation of frontal brain area of a skilled chess player is lower than that of a lower-skilled player, which suggests that training or practice reduces the need for frontal executive functions since information processing in the posterior regions becomes more automated^55^. These previous investigations reveal that ERD in the frontal upper alpha band reflects mental operation of information and the executive control of cognitive processing. We speculate that the stronger frontal upper alpha ERD is indicative of mental effort exerted by higher executive function and working memory demands to reason the high complexity scientific problems. For these problems, students not only require more mental effort on mental operations of interactive information, but also need more anterior control over relative posterior brain regions responsible for specific cognitive processes.

In summary, we investigated online mental effort in solving scientific problems using the EEG. We suggest that the increase in the frontal theta activity and decrease in the anterior upper alpha activity reflect the mental effort allocated by working memory and cognitive control. The decrease in the centro-parietal upper alpha activity reflects the mental effort imposed by visuospatial processing and magnitude manipulation of information in the scientific problems. The decrease in the upper alpha activity in the occipital area reflects the stimulus encoding and semantic processing of scientific conceptions. The task-induced EEG changes suggest the mental resources required for the specific cognitive processes involved in solving scientific problems. It can provide a deep understanding of students’ learning outcomes. It will also provide implications for the development of effective guidance to improve their ability to solve scientific problems in educational practice.

## Methods

### Participants

Twenty-five healthy students majoring in engineering or science (mean age = 23 ± 1.8 years old, 12 males and 13 females) from Southeast University participated in this study. No physics department students were recruited. All of the participants were right-handed, had normal or correct-to-normal vision, reported no history of psychiatric or neurological disorders, and were not taking any medications. Each participant signed a written informed consent before the experiment and received monetary compensation for participation. Ethical approval for the study was obtained from the ethics committee of Southeast University. All procedures were conducted in accordance with approved guidelines and regulations.

### Scientific problems

The scientific problems used in our study are items to analyze the forces acting on objects in various states of motion. Force analysis plays an important role in science learning and is a fundamental step in solving many physics problems. However, it is usually a challenging for students to conduct force analysis, especially for the objects in complex motion state. To solve these problems, students required to analyze the interaction between objects, retrieve scientific conceptions about the relationship between force and motion, as well as make inferences about presented information based on these conceptions. Cognitive resources across various brain regions were recruited and task-related mental effort were allocated. As widely used in science education practice, the problems used in our study were presented as the symbolic diagrams. These symbolic diagrams conveyed precise meanings and combined with rules of force and motion that must be used correctly^67^.

**Figure 3:**
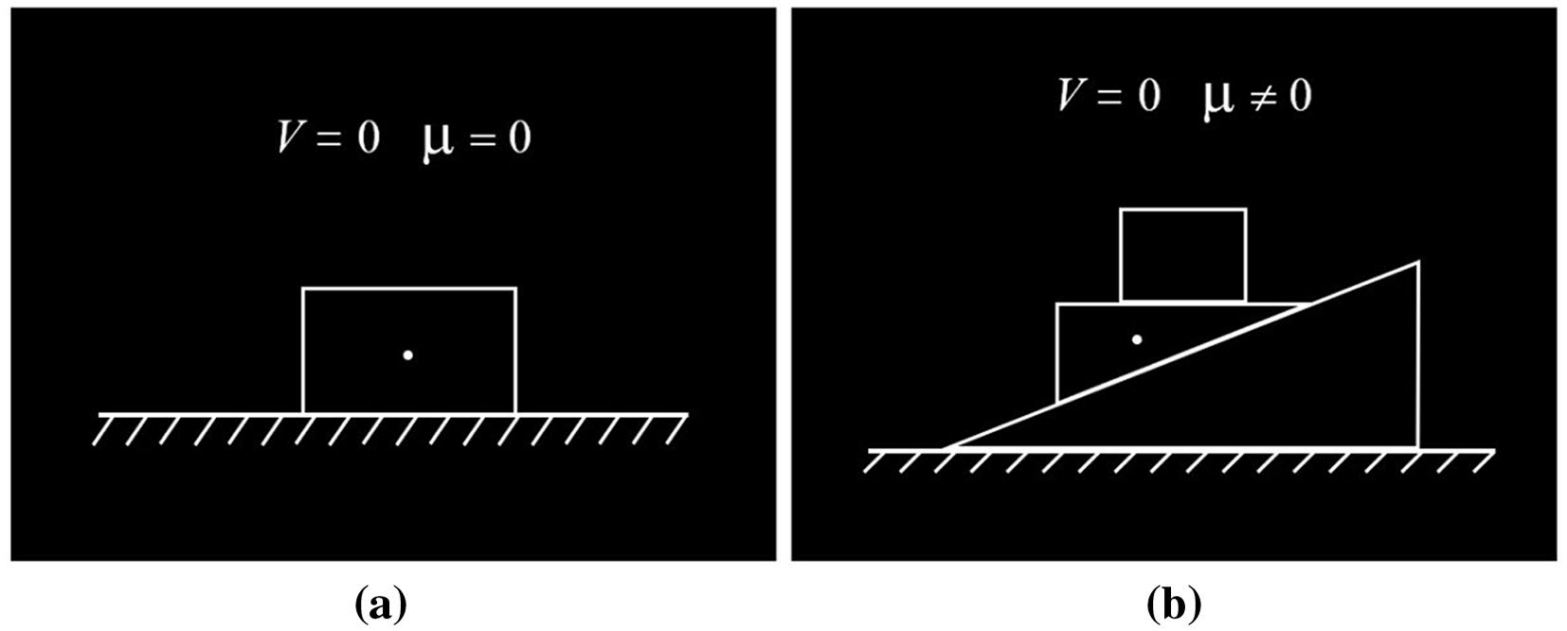
Examples of scientific problems presented in the task. Problems are illustrated as the symbolic diagrams commonly used in science education practice. Students are asked to determine the number of forces acting on the object marked by the small white dot. The velocity of the object (*V*) and the friction of contacting surfaces (***μ***) are shown in the diagram. (**a**) Low complexity scientific problem. (**b**) High complexity scientific problem.

The whole task consisted of 24 scientific problems which were divided into two complex conditions. For the low complexity problems, only one object was considered. The forces acting on the object would be gravity, friction depending on the roughness of the contact surface, the supporting force of the surface, and the pulling or pushing force applied on it if present. For the high complexity problems, two or more objects were involved, so the interaction between the objects had to be carefully analyzed. Typical examples of the scientific problems in the low and high complexity conditions are illustrated in Fig. 3. The final 24 scientific problems were selected from a bank of 100 force analysis items. The evaluation procedure was conducted by a committee composed of three experienced physics teachers and two university professors from the physics department to ensure the validity of the complexity classification. In the experiment, the students were asked to determine the total amount of force acting on an object. We assumed that the high complexity problems were more demanding and required higher mental effort. We also expected that the different amounts of online mental effort in solving scientific problems would be reflected by EEG signals. In the experiment, we also developed items as controls, in which students were only required to count the number of objects in a particular shape.

### Experimental procedures

**Figure 4:**
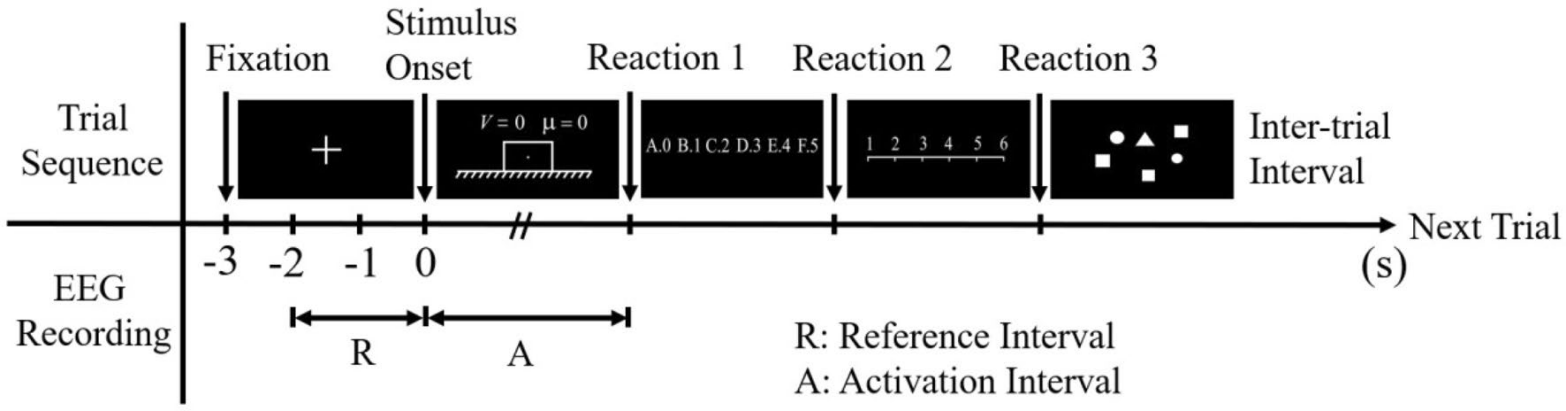
Schematic of a single trial. The time period from the onset of a scientific problem to reaction 1 serves as the activation interval, the time period from 1 to 3 s after the onset of the fixation cross serves as the reference interval.

A total of 48 stimuli including 24 scientific problems and 24 control items were presented using E-prime 2.0. The stimuli were presented according to the event-related design, as shown in Fig. 4. Each trial started with the presentation of a central white fixation cross on the black screen for 3000 ms. Then a scientific problem was presented and the participants were required to think silently about the forces acting on the object which was marked with a small white dot. The problem remained on the screen until the participant got the answer and requested the answer options by keystroke (Reaction 1). After that, the scientific problem disappeared from the screen and the answer options were presented. The participant selected the answer from six options by pressing the corresponding reaction button (Reaction 2). Finally, a subjective rating scale was presented to assess the level of effort put into the problem, and the participant responded by pressing the respective button (Reaction 3). After the scientific problem was presented, a control item was showed. An inter-trial interval of 3000 ms was designed as a resting period before presenting the next trial. The purpose of the application of control stimuli was to reset the mental activity of the participant to normal levels and eliminate any stressful effects of the previous scientific problem. Only EEG signals during solving scientific problems were analyzed.

The participants sat comfortably about 75 cm away from the screen to perform the task and were asked to pay attention to the task while keeping still. Two scientific problems of low and high complexity were used in the practice session for the familiarity of the experiment. The remaining problems were divided into two blocks in the formal experiment procedure. The scientific problems of varying levels of complexity were randomly presented and equally distributed between each block. The participants’ EEG signals in the task were recorded at the same time. After the EEG recording session, participants were interviewed to explain how they solved each scientific problem and how hard they worked on it during the task.

### Behavioral data analysis

The behavioral data including reaction time, response accuracy and subjective effort evaluation were recorded by E-prime software. In our study, the reaction time was identified as the time interval between onset of scientific problem and their keystroke for reaction 1. The correctness of response to each question is determined according to their keystroke of answer options for Reaction 2. Subjective effort evaluation was obtained according to their keystroke of Reaction 3. The behavioral data for each student was first averaged for problems in the low and high complexity respectively. Then, the mean and standard deviation of each behavioral data were calculated across all students for each problem condition.

We first performed the Shapiro–Wilk normality test for each behavioral data. It revealed that the data did not conform to a normal distribution (all *p* < 0.05). Accordingly, we conducted Wilcoxon signed-rank test to analyze the main effect of problem complexity condition on each behavioral data.

We further compared behavioral data between correct and incorrect trials for high complexity problems. For reaction time of correct and incorrect responses, wilcoxon signed-rank test revealed that reaction time of incorrect trials was comparable to that of correct trials (*Z* = − 0.44, *p* = 0.67). The same analysis was conducted to subjective evaluation of mental effort, which also showed no significant difference between two kinds of conditions (*Z* = − 0.97, *p* = 0.33). Additionally, in the interview session, the students also expressed that the amount of effort to solve these difficult problems was high no matter they successfully solved it or not. All these results suggested that the students spent a similar amount of effort to solve the high complexity problems even though they may not obtain the right answer for certain problem. For this reason, we included all trials for further EEG data analysis.

### EEG recording and data analysis

EEG was recorded from 32 tin electrodes mounted on an elastic cap according to the international 10–20 system (NeuroScan Inc., Herndon, VA, USA). The electrooculography (EOG) was recorded from two electrodes on the canthi and two electrodes located above and below the right eye. All electrode impedances were maintained below 5 KΩ. EEG and EOG signals were continuously sampled at 500 Hz for off-line analysis. The EEG data were band-pass filtered between 0.5 Hz and 60 Hz. Ocular artifacts were first corrected with an eye-movement correction algorithm which employed a regression analysis in combination with artifact averaging^68^. The continuous EEG data were segmented into epochs covering reference interval and activation interval in each trial. To further remove possible artifacts, the data were submitted to Independent Component Analysis (ICA) using the runica function from the EEGLAB toolbox to clear visible artifacts, such as the components of possible ocular and muscle movements.

Considering that brain activity varies individually, event-related synchronization/ desynchronization (ERS/ERD) of EEG signals was quantified to measure cortical activation and topographical distribution in scientific problem-solving^52^. The amount of ERS/ERD at a given frequency band is defined as the percentage of power increase/decrease during the activation interval relative to the reference interval. The activation interval refers to the time period while working on a task, and the reference interval refers to a pre-stimulus time period without any task demands. In our study, the time period from the onset of a scientific problem to reaction 1 served as the activation interval, and time period from 1 to 3 s after the onset of the fixation cross served as the reference interval, as shown in Fig. 4.

**Figure 5:**
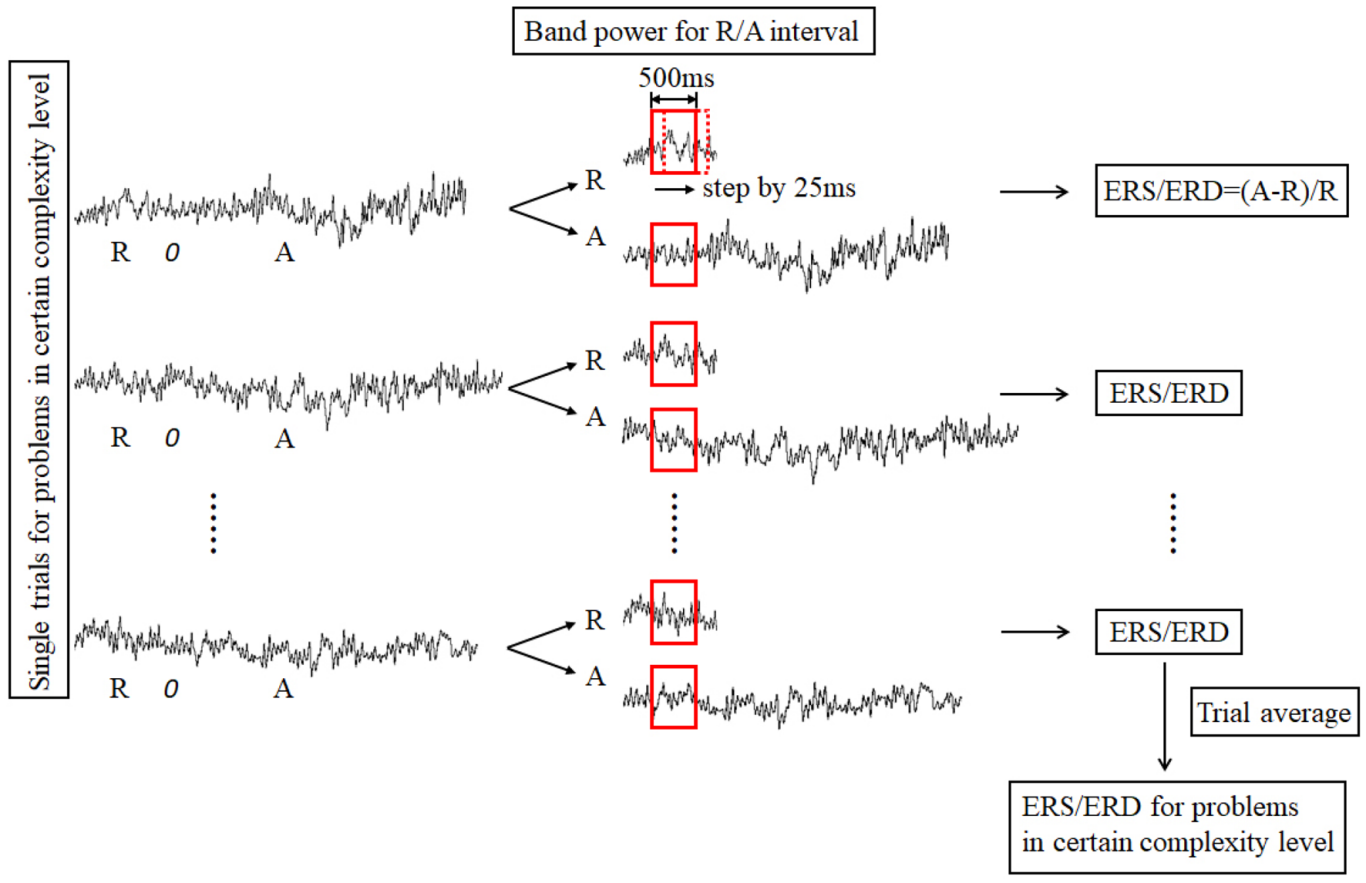
Steps of EEG signal processing to calculate ERS/ERD values.

**Figure 6:**
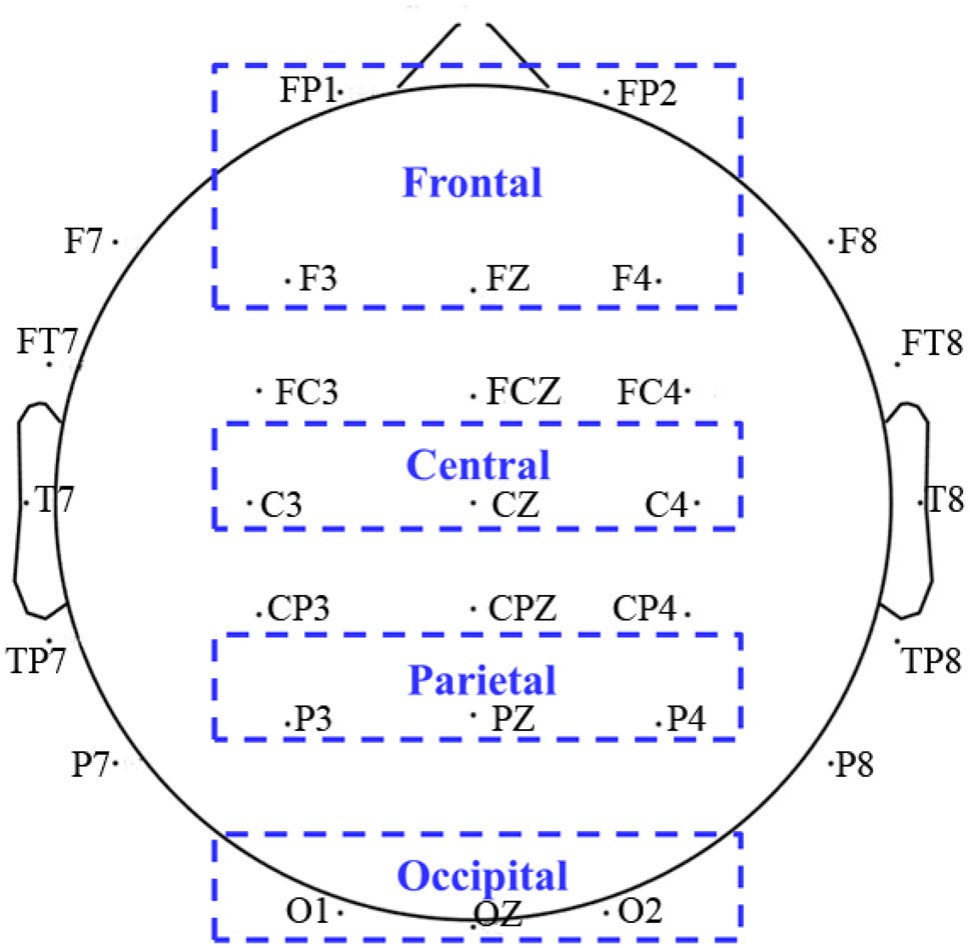
Division of the brain regions and their included electrodes for ERS/ERD calculation. Frontal (FP1, FP2, F3, Fz and F4), Central (C3, Cz and C4), Parietal (P3, Pz, and P4) and Occipital (O1, Oz, and O2).

Figure 5 illustrates the steps of EEG signal processing. For each epoch, EEG time series in the reference and activation intervals were converted into the frequency domain using a fast Fourier transformation (FFT) with a sliding 500 ms window by a step of 25 ms. Therefore, each window overlapped the previous one by 475 sample points. Band power in theta (4–7 Hz), lower alpha (8–10 Hz) and upper alpha (10–12 Hz) frequencies were calculated, respectively. The percentage change in the frequency band power from the activation interval to reference interval in each trial was then quantified. Finally, the calculated change in band power was averaged across all trials for low and high complexity conditions respectively. A positive value represents ERS and a negative value suggests ERD. For statistical analyses, ERS/ERD data were aggregated over different electrode locations as shown in Fig. 6: frontal (FP1, FP2, F3, Fz and F4), central (C3, Cz and C4), parietal (P3, Pz, and P4) and occipital (O1, Oz, and O2).

For statically analysis, problem complexity condition (low and high complexity) and brain area (frontal, central, parietal and occipital areas) are two within-subjects variables. Therefore, we first conducted the Shapiro–Wilk normality test on each set of EEG data. The statistical results revealed that each set of EEG data was normally distributed (all *p* > 0.05). Then, we performed the two-way repeated measures ANOVA on ERS/ERD data to analyze the main and interaction effects of problem complexity and brain area. Greenhouse–Geisser correction was applied to correct for violations of the sphericity assumption, all post-hoc tests were bonferroni-corrected.
